# Heterogeneous SARS-CoV-2 humoral response after COVID-19 vaccination and/or infection in the general population

**DOI:** 10.1038/s41598-022-11787-4

**Published:** 2022-05-21

**Authors:** Fabrice Carrat, Paola Mariela Saba Villarroel, Nathanael Lapidus, Toscane Fourié, Hélène Blanché, Céline Dorival, Jérôme Nicol, Jean-François Deleuze, Olivier Robineau, SAPRIS-SERO Study Group, Fabrice Carrat, Marie Zins, Gianluca Severi, Mathilde Touvier, Hélène Blanché, Jean-François Deleuze, Xavier de Lamballerie, Clovis Lusivika-Nzinga, Gregory Pannetier, Nathanael Lapidus, Isabelle Goderel, Céline Dorival, Jérôme Nicol, Olivier Robineau, Sofiane Kab, Adeline Renuy, Stéphane Le-Got, Céline Ribet, Mireille Pellicer, Emmanuel Wiernik, Marcel Goldberg, Fanny Artaud, Pascale Gerbouin-Rérolle, Mélody Enguix, Camille Laplanche, Roselyn Gomes-Rima, Lyan Hoang, Emmanuelle Correia, Alpha Amadou Barry, Nadège Senina, Julien Allegre, Fabien Szabo de Edelenyi, Nathalie Druesne-Pecollo, Younes Esseddik, Serge Hercberg, Mélanie Deschasaux, Hélène Blanché, Jean-Marc Sébaoun, Jean-Christophe Beaudoin, Laetitia Gressin, Valérie Morel, Ouissam Ouili, Jean-François Deleuze, Laetitia Ninove, Stéphane Priet, Paola Mariela Saba Villarroel, Toscane Fourié, Souand Mohamed Ali, Abdenour Amroun, Morgan Seston, Nazli Ayhan, Boris Pastorino, Mathilde Touvier, Gianluca Severi, Marie Zins, Xavier de Lamballerie

**Affiliations:** 1 Institut Pierre-Louis d’Épidémiologie et de Santé Publique, Sorbonne Université, Inserm, Département de santé publique, Hôpital Saint-Antoine, APHP, 27 rue Chaligny, 75571 Paris Cedex 12, France; 2 Unité des Virus Émergents, UVE, IRD 190, INSERM 1207, Aix Marseille Univ, IHU Méditerranée Infection, Marseille, France; 3 Fondation Jean Dausset-CEPH (Centre d’Etude du Polymorphisme Humain), CEPH-Biobank, Paris, France; 4 Institut Pierre-Louis d’Épidémiologie et de Santé Publique, Sorbonne Université, Inserm, Paris, France; 5 Inserm U1153, Inrae U1125, Cnam, Nutritional Epidemiology Research Team (EREN), Sorbonne Paris Nord University, Epidemiology and Statistics Research Center – University of Paris (CRESS), Bobigny, France; 6 CESP UMR1018, UVSQ, Inserm, Université Paris-Saclay, Gustave Roussy, Villejuif, France; 7 Department of Statistics, Computer Science and Applications, University of Florence, Florence, Italy; 8 Paris University, Paris, France; 9 UVSQ, Inserm UMS 11, Université Paris-Saclay, Université de Paris, Villejuif, France

**Keywords:** Viral infection, Risk factors

## Abstract

Assessment of the intensity, dynamics and determinants of the antibody response after SARS-CoV-2 infection or vaccination in the general population is critical to guide vaccination policies. This study characterized the anti-spike IgG titers in 13,971 participants included in a French multicohort population-based serological survey on COVID-19 between April and October 2020 and followed-up with serological testing between May and October 2021. Eight follow-up profiles were defined depending on SARS-CoV-2 infection (0, 1 or 2) and COVID-19 vaccination (0, 1, 2 or 3). The anti-spike titer was lower in adults with no vaccination even in case of infection or reinfection, while it was higher in adults with infection followed by vaccination. The anti-spike titer was negatively correlated with age in vaccinated but uninfected adults, whereas it was positively correlated with age in unvaccinated but infected adults. In adults with 2 vaccine injections and no infection, the vaccine protocol, age, gender, and time since the last vaccine injection were independently associated with the anti-spike titer. The decrease in anti-spike titer was much more rapid in vaccinated than in infected subjects. These results highlight the strong heterogeneity of the antibody response against SARS-CoV-2 in the general population depending on previous infection and vaccination.

## Introduction

Since December 2019, the world has been experiencing the COVID-19 pandemic, and thanks to a historically unprecedented effort, vaccines were developed in less than one year and have been used for mass vaccination since December 2020. In December 1, 2021 more than 7.7 billion doses had been administered worldwide^1^. In France, vaccination against SARS-CoV-2 began on December 27, 2020, first for individuals living in nursing homes and healthcare professionals in contact with these patients, then gradually to the entire population aged 12 years or older, by June 2021^2^. Three vaccines have mainly been used, BNT162b2 (Pfizer-BioNTech, PFI), mRNA-1273 (Moderna, MOD) and ChAdOx1 nCoV-19 (Astra-Zeneca, AST) while the Ad26.COV2.S (Janssen) vaccine has been authorized since mid-March 2021. On December 1, 2021, 89.3% of the French adult population had received two-doses of SARS-CoV-2 vaccines^3^.

Numerous studies have shown a progressive decrease in antibodies induced by vaccination^4–6^ or natural infection^7,8^, in particular neutralizing antibodies. Correlates of protection have been established^9–11^. An assessment of the serological status of the general population and the factors associated with the level of antibody titers considered to be protective are critical to guide vaccination policy.

Our objective was to characterize the humoral status of participants from general population-based cohorts in relation to vaccination or infection or both, and to explore the associated factors.

## Methods

### Design, participants and methods

We used data from the SAPRIS (“SAnté, Perception, pratiques, Relations et Inégalités Sociales en population générale pendant la crise COVID-19”)—SERO survey in France. The study has been described elsewhere^12,13^. It is based on a consortium of prospective cohort studies (Constances, E3NE4N and Nutrinet-Santé) in the general population including 279,478 adult volunteers with regular access to electronic (internet) questionnaires.

Two self-administered questionnaires covering the first wave of the pandemic were sent as of April 1, 2020 and returned before May 27, 2020. The questionnaires included socio-demographics, household size and composition, history of COVID-19 diagnosis and SARS-CoV-2 RT-PCR testing (information on whether or not the RT-PCR performed was quantitative was not collected, so the term RT-PCR is used for qualitative RT-PCR or RT-qPCR throughout the manuscript), a detailed description of the participant’s symptoms in the previous weeks, and an invitation to perform a serology by self-sampling dried-blood spot (DBS). Participants living in mainland France who completed the questionnaires and who agreed to the serology received a DBS kit to be returned to the centralized biobank between May and November, 2020, after capillary blood collection (CEPH Biobank, Paris, France). The Elisa test (Euroimmun^®^, Lübeck, Germany) was used to detect anti-SARS-CoV-2 antibodies (IgG) directed against the S1 domain of the spike protein of the virus. In accordance with the manufacturer’s instructions, a test was considered to be positive with an optical density ratio (ODR) ≥ 1.1, indeterminate between 0.8 and 1.1, and negative, < 0.8. All samples with a ODR ≥ 0.7 were also tested with an in-house micro-neutralization assay to detect neutralizing anti-SARS-CoV-2 antibodies (SN), as described elsewhere, with a positive SN defined as a titer ≥ 40^14^. The reported sensitivity and specificity of the anti-spike IgG test at the 1.1 threshold (considering indeterminate results as negative) is 87% and 97.5%, respectively^15^. More details on serological methods can be found in Ref.^12^. At the end of the first wave of the pandemic, 100,719 participants living in mainland France had completed the two baseline questionnaires (overall participation rate, 36%) and 82,521 had performed a serology (93% of those invited). A total of 77,580 of these participants had a baseline serology result and had completed 2 questionnaires: 3433 (4.4%) had a positive anti-spike IgG test, 1233 (1.6%) had an indeterminate test and 72,914 (94.0%) had a negative test.

A follow-up self-questionnaire was received from all participants between June 2, 2021 and October 21, 2021. The questionnaire included detailed information on vaccination (full description of the vaccine protocol, including the type of vaccine, dates of injection, except in the E3N and E4NG1 cohorts where only the last injection was described), diagnosis of SARS-CoV-2 infection, symptoms and healthcare use since the baseline questionnaires. Altogether, 56,064 (72%) participants completed the follow-up questionnaire. All participants with a positive or indeterminate baseline ELISA-S test, a positive SN result or who reported a positive dated diagnosis of SARS-CoV-2 infection by RT-PCR (n = 4755) and a random sample of 11,000 participants with a negative baseline anti-spike IgG test result and no diagnosis of SARS-CoV-2 infection during the first wave of the pandemic were also invited to perform a follow-up serology by DBS. The anti-spike IgG test was used to evaluate serological status at follow-up using the same positivity threshold as at baseline and with a maximum value of 13 ODR. Detection of neutralizing anti-SARS-CoV-2 antibodies was not available on these follow-up samples. In participants with a positive anti-spike IgG test, antibody concentrations were expressed in units per milliliter and converted to binding antibody units (BAU), using the conversion factor (3.2) recommended by the manufacturer^16^. Samples with values > 384 BAU/mL were diluted 1:20 allowing a range extension up to 7680 BAU/mL.

### Outcomes

Our main outcome was the anti-spike IgG result on the follow-up sample. An anti-spike IgG titer ≥ 264 BAU/mL was considered to be associated with 80% vaccine efficacy against symptomatic infection to the Alpha (B.1.1.7) variant^9^ and was used to divide participants into two groups. Participants with negative or indeterminate anti-spike IgG test results were considered to have a standardized titer below 264 BAU/mL.

### Covariate definitions

Participants infected during the first wave were defined as those who reported a positive RT-PCR dated before the first DBS, or with a positive anti-spike IgG test or positive SN at the first sampling. Eight follow-up profiles were defined according to whether the participant had been vaccinated at the follow-up sample (0: NoVac, 1: 1Vac or 2/3: 2/3Vac doses, with 14 days between the last vaccine dose and DBS for a dose to count) and whether the participant had been infected during the first wave of the pandemic or had received a RT-PCR-confirmed diagnosis of SARS-CoV-2 infection during follow-up (NoDiag: neither infection nor diagnosis, 1Diag: either infection or diagnosis, 2Diag: infection during the first wave and diagnosis during follow-up).

### Statistics

The anti-spike IgG value (in ODR or BAU/mL) was log-transformed in all quantitative analyses. In a first analysis, we explored the factors associated with the value of anti-spike IgG (in ODR) in all participants with available follow-up serological results. We used a generalized linear model with Bonferroni adjustments for multiple comparisons to compare the log-transformed anti-spike IgG value between groups. The Spearman correlation coefficient was used to test associations between age and anti-spike IgG in groups. To explore the longitudinal dynamics of anti-spike IgG (ODR) in participants infected during the first wave of the pandemic, we used a linear mixed-model as well as a power-law model^17^ with random per participant parameters. Locally weighted polynomial smoothing (LOESS) was used to explore the relationship between the log-transformed anti-spike IgG value at follow-up and the time since the last vaccine injection. Slope estimates of the decrease in log-titers with the time since the last injection were performed by generalized linear modelling adjusted for age, gender, chronic diseases, BMI and time between the first and second vaccine injection. Comparisons of slopes between vaccine protocols were performed by testing the interaction term between the vaccine protocol and time since the last injection. We used logistic regression to identify factors associated with anti-spike IgG ≥ 264 BAU/mL (versus < 264 BAU/mL) in participants with 2 vaccine injections and no diagnosis of a SARS-CoV-2 infection. Age, time since the last injection and time between the first and second vaccine injection were entered as continuous covariates into the logistic model and linearity was checked by comparing the model estimation using a single linear regression coefficient with the model using a thin-plate regression spline for the tested covariate (based on the Akaike Information Criteria). All covariates significantly associated with anti-spike IgG ≥ 264 BAU/mL in univariable analysis were included in the multivariable model. We did not use imputation methods for missing covariates because analysis of all cases represented 96.0% of the original dataset. All statistical tests were two-tailed with a type I error of 5%. Statistical analysis was performed using SAS v9.4 software^®^ (SAS Institute, Cary, NC, USA).

### Ethics approval/consent to participate

Ethical approval and written or electronic informed consent were obtained from each participant before enrolment in the original cohort. The SAPRIS-SERO study was approved by the Sud-Mediterranée III ethics committee (approval #20.04.22.74247) and electronic informed consent was obtained from all participants for DBS testing. The study was registered (#NCT04392388). All methods were performed in accordance with the relevant guidelines and regulations.

## Results

Of 15,755 participants who were invited to perform a follow-up serology, 14,968 returned the DBS, 13,971 (88%) had interpretable serologic results as well as a follow-up questionnaire and were evaluated in this analysis.

Participant characteristics are described in Supplementary Table S1. Participants’ median age was 58 (Q1–Q3: 45–71) years, with 66% women. A total of 2913 participants (20.9%) were infected during the first wave of the pandemic (2275 anti-spike IgG positive, 963 SN positive, 212 positive dated RT-PCR). The follow-up sample was collected a median of 330 (Q1–Q3: 317–358) days and the follow-up questionnaire a median of 332 (Q1–Q3: 317–373) days after the initial sample. Overall, 743 (5.3%) participants reported a positive diagnosis of SARS-CoV-2 infection during follow-up, a median of 182 (Q1–Q3: 104–245) days before follow-up serology, 149 in 2913 participants who had been infected during the first wave of the pandemic (5.1%) and 594 in 11,058 participants with no infection during the first wave (5.4%). Three thousand one hundred thirty (22.4%) participants remained unvaccinated while 5088 (36.4%), 5725 (41.0%) and 28 (0.2%) had received one, two or three vaccine injections, respectively. The first, second and third vaccine doses were received a median of 60 (Q1–Q3: 39–88), 55 (35–95) and 35 (26–45) days before the follow-up serology, respectively.

**Figure 1:**
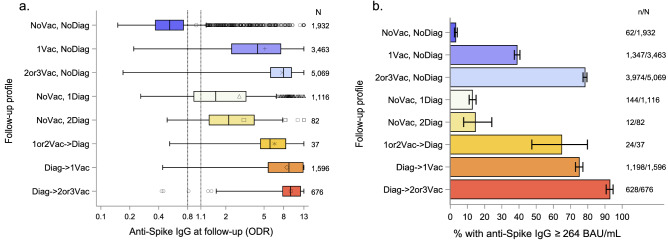
Distribution (boxplot) of anti-spike IgG (ODR) according to follow-up profiles. (**a**) Anti-spike IgG (ODR). The dashed lines show the threshold values for a positive (≥ 1.1), indeterminate [0.8–1.1[ or negative (< 0.8) test result. (**b**) Proportion of participants with anti-spike IgG ≥ 264 BAU/mL according to follow-up profiles. Error bars represent 95% Confidence Intervals calculated using an exact method.

**Figure 2:**
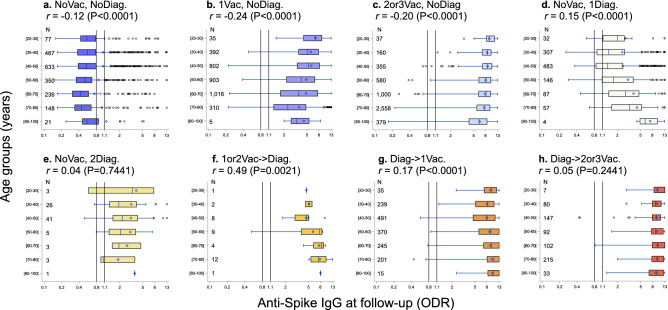
Distribution (boxplot) of anti-spike IgG (ODR) according to age groups by follow-up profiles (**a–h**). The dashed lines show the threshold values for a positive (≥ 1.1), indeterminate [0.8–1.1[ or negative (< 0.8) test result. Spearman correlation coefficients between age and log-IgG titer are presented.

The follow-up profiles and the corresponding distributions of anti-spike IgG (ODR) are described in Table 1 and Fig. 1. The mean log-IgG values were different in all pairwise comparisons of follow-up profiles (Bonferroni adjusted P < 0.05) except between the (1or 2Vac- > Diag) versus (2 or 3Vac, NoDiag) (P = 0.9842) and (Diag- > 1Vac) (P = 0.1702) subgroups and between the (NoVac, 1Diag) and the (NoVac, 2Diag) subgroups (P = 0.0717) (Fig. 1a). The proportion of participants with anti-spike IgG ≥ 264 BAU/mL was 3.2% (95% CI 2.5%; 4.1%) in the (NoVac, NoDiag), 39% (95% CI 37%; 41%) in the (1Vac, NoDiag), 78% (95% CI 77%; 80%) in the (2 or 3Vac, NoDiag), 13% (95% CI 11%; 15%) in the (NoVac, 1Diag), 15% (95% CI 8%; 24%) in the (NoVac, 2Diag), 65% (95% CI 47%; 80%) in the (1 or 2Vac- > Diag), 75% (95% CI 73%; 77%) in the (Diag- > 1Vac) and 93% (95% CI 91%; 95%) in the (Diag- > 2or3Vac), P < 0.0001 (Fig. 1b, Supplementary Table S2). The association of anti-spike IgG distribution (ODR) varied with age (Fig. 2). The correlation with age was negative when there was no SARS-CoV-2 infection during the first wave or diagnosis during follow-up, while it was positive in participants with an infection or diagnosis, except in reinfected participants (NoVac, 2Diag) as well as in participants with two vaccine injections after infection or diagnosis (Diag- > 2or3Vac). The relationships between age and follow-up profiles were similar in the proportion of participants with anti-spike IgG ≥ 264 BAU/mL (see Supplementary Fig. S1). The decrease in the anti-spike IgG over time was relatively slow in both linear and non-linear mixed model estimates in 834 participants infected during the first wave of the pandemic who were unvaccinated at the follow-up serology (see Supplementary Fig. S2). However, only 514 (64%, 95% CI (61%; 68%)) of 801 remained anti-spike IgG positive (ODR) at the follow-up sample and only 74 (8.9%, 95% CI (7.0%; 11%)) had anti-spike IgG ≥ 264 BAU/mL. Although the anti-spike IgG titer was lower with one than with two vaccine injections in participants who were vaccinated after infection or a diagnosis (difference between 1 versus 2 vaccine injections =  − 0.31, 95% CI − 0.45; − 0.19) log(BAU/mL), the decrease over time since the last injection was not significant with one injection (adjusted slope estimate = 0.06, 95% CI − 0.01; 0.14) log(BAU/mL) for every 4 weeks, while it was − 0.24 (95% CI − 0.18; − 0.30) log(BAU/mL) for every 4 weeks after the second injection (see Supplementary Fig. S3).

**Table 1:** Follow-up profiles.

Follow-up profiles	n = 13,971
**No vaccination, no diagnosis of SARS-CoV-2 infection (NoVac, NoDiag)**	1932 (13.8%)
**1 vaccine injection, no diagnosis of SARS-CoV-2 infection (1Vac, NoDiag)**	3463 (24.8%)
**2 or 3 vaccine injections, no diagnosis of SARS-CoV-2 infection (2or3Vac, NoDiag)**^a^	5069 (36.3%)
**No vaccination, diagnosis of SARS-CoV-2 infection (NoVac, 1Diag)**	1116 (8.0%)
Infected during the first pandemic wave	834
Infected during the follow-up	282
**No vaccination, 2 diagnoses of SARS-CoV-2 infection (NoVac, 2Diag)**	82 (0.6%)
Infected during the first pandemic wave	82
Infected during the follow-up	82
**1 or 2 vaccine injections before a diagnosis of SARS-CoV-2 infection (1or2Vac- > Diag)**^**b**^	37 (0.3%)
Infected during the first pandemic wave	4
Infected during the follow-up	37
**Diagnosis of SARS-CoV-2 infection before 1 vaccine injection (Diag- > 1Vac)**	1596 (11.4%)
Infected during the first pandemic wave	1350
Infected during the follow-up	303
**Diagnosis of SARS-CoV-2 infection before 2 or 3 vaccine injection (Diag- > 2or3Vac)**^**c**^	676 (4.8%)
Infected during the first pandemic wave	643
Infected during the follow-up	39

^a^23 received 3 vaccine injections.

^b^29 received 1 vaccine injection, 8 received 2 vaccine injections.

^c^5 received 3 vaccine injections.

**Figure 3:**
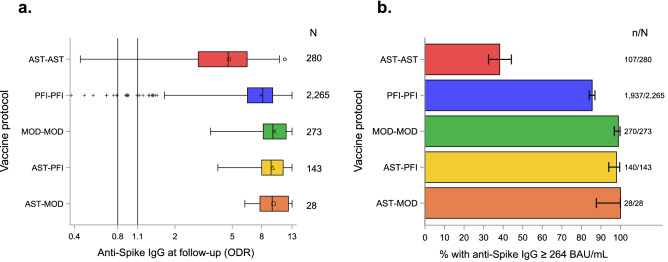
Distribution (boxplot) of anti-spike IgG according to vaccine protocol**.** Abbreviations for first and second vaccine doses are AST = ChAdOx1 nCoV-19; MOD = mRNA-1273; PFI = BNT162b2. (**a**) Anti-spike IgG (ODR). The dashed lines show the threshold values for a positive (≥ 1.1), indeterminate [0.8–1.1[ or negative (< 0.8) test result. (**b**) Proportion of participants with anti-spike IgG ≥ 264 BAU/mL according to the vaccination protocol. Error bars represent 95% Confidence Intervals calculated using an exact method.

**Figure 4:**
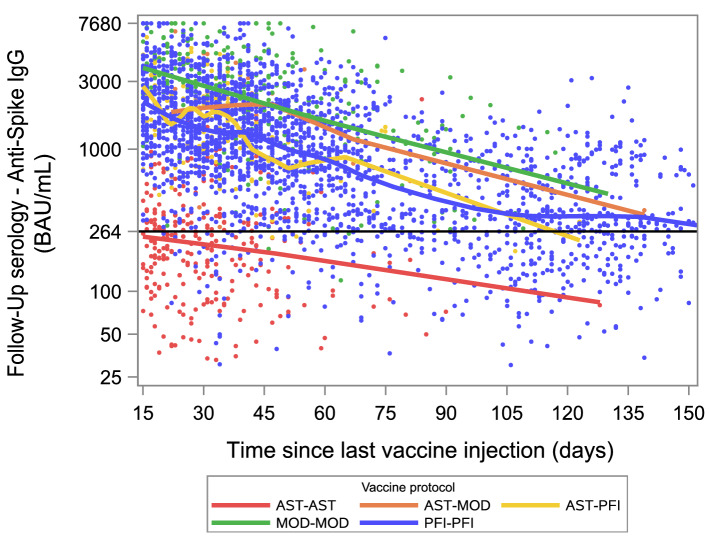
Scatter plot of anti-spike IgG (BAU/mL) according to time since the second vaccine dose by vaccine protocol, and locally weighted polynomial smoothing (LOESS) trend estimates. Abbreviations for first and second vaccine doses are AST = ChAdOx1 nCoV-19; MOD = mRNA-1273; PFI = BNT162b2. The dashed line at 264 BAU/mL was estimated to be associated with 80% vaccine efficacy against symptomatic infection with the Alpha (B.1.1.7) variant^9^. Seventeen samples not shown.

Two thousand nine hundred eighty-nine participants with 2 vaccine injections and no diagnosis of SARS-CoV-2 infection were selected for the second analysis. Twenty-three participants who received 3 vaccine injections, 1995 participants from the E3N and E4NG1 cohorts and another 62 participants with missing data on vaccine type or vaccine date were excluded: 2964 (99.2%, 95% CI (98.8%; 99.5%)) had a positive anti-spike IgG (ODR) and 2482 (83.0%, 95% CI (81.6%; 84.4%)) had an anti-spike IgG ≥ 264 BAU/mL. The median time since the last injection was 44 (Q1–Q3: 29–72) days and the median time between the two vaccine doses was 28 (Q1–Q3: 27–40) days. There was a strong association between the anti-spike IgG level (in ODR or BAU/mL) and the vaccine protocol with lower titers in participants vaccinated with two doses of ChAdOx1 nCoV-19 (AST-AST, n = 280), compared to two-doses of BNT162b2 (PFI-PFI, n = 2265) (P < 0.0001), and lower titers in the latter group compared to two-doses of mRNA-1273 (MOD-MOD, n = 273) (P < 0.0001) or the combinations of one dose of ChAdOx1 nCoV-19 with one dose of BNT162b2 (AST-PFI, n = 143) (P < 0.0001) or with one dose of mRNA-1273 (AST-MOD, n = 28) (P = 0.0290 for anti-spike IgG in ODR, P = 0.0012 for anti-spike IgG in BAU/mL) (Fig. 3a). Similar associations were observed for the proportion of participants with anti-spike IgG ≥ 264 BAU/mL (Fig. 3b) The decrease in the log-titer was linearly associated with time since the last injection (Fig. 4), with an adjusted slope estimate of − 0.46 (95% CI − 0.48; − 0.43) log(BAU/mL) for every 4 weeks after the second vaccine injection. The slopes did not differ significantly between the vaccine protocols (P = 0.0674).

The vaccine protocol, age, gender, and time since the last vaccine injection were independently associated with the probability of anti-spike IgG ≥ 264 BAU/mL in the follow-up sample (Table 2).

**Table 2:** Factors associated with an anti-spike IgG titer ≥ 264 BAU/mL at the follow-up in participants with two vaccine doses and no diagnosis of SARS-CoV-2 infection.

	Anti-spike IgG ≥ 264 BAU/mL n/N (%)	Univariable odds-ratio (OR (95%CI))	P-value^a^	Multivariable adjusted odds-ratio (95%CI)^b^	P-value^a^
**Age group**		Per 10 years increase	< 0.0001	Per 10 years increase	< 0.0001
[20–30]	35/37 (95)	0.78 (0.72;0.85)		0.79 (0.70;0.88)	
[30–40]	136/153 (89)				
[40–50]	313/342 (92)				
[50–60]	457/555 (82)				
[60–70]	771/947 (81)				
[70–80]	720/883 (82)				
80+	50/72 (69)				
**Gender**			< 0.0001		0.0016
Male	920/1161 (79)	Ref		Ref	
Female	1562/1828 (85)	1.54 (1.27; 1.87)		1.49 (1.16; 1.91)	
**BMI (kg/m**^**2**^**)**			0.0165		0.5143
< 25	1465/1736 (85)	Ref		Ref	
[25; 30] (overweight)	735/896 (82)	0.84 (0.68; 1.05)		1.13 (0.86; 1.47)	
≥ 30 (obese)	226/290 (78)	0.65 (0.48; 0.89)		0.91 (0.61; 1.34)	
Missing	67				
**Smoking status**			0.1414		
Non smoker	1069/1264 (85)	Ref		–^c^	–
Active smoker	232/282 (82)	0.85 (0.60; 1.19)			
Ex-smoker	1136/1390 (82)	0.82 (0.67; 1.00)			
Missing	53				
**Alcohol use (in g/day)**			0.1297		
< 5	947/1111 (85)	Ref		–^c^	–
[5,10]	441/536 (82)	0.80 (0.61; 1.06)			
[10,20]	530/644 (82)	0.81 (0.62; 1.05)			
[20,30]	240/300 (80)	0.69 (0.50; 0.96)			
≥ 30	166/206 (81)	0.72 (0.49; 1.05)			
Missing	192				
**Chronic diseases**			0.0052		0.4690
No	1508/1780 (85)	Ref		Ref	
Yes	952/1184 (80)	0.74 (0.61; 0.90)		0.88 (0.69; 1.11)	
Don’t know	15/16 (94)	2.70 (0.36; 20.5)		1.76 (0.21; 14.7)	
Missing	9				
Time between since last vaccine injection (per 4 weeks increase)		0.63 (0.59; 0.67)	< 0.0001	0.43 (0.39; 0.47)	< 0.0001
Time between the two vaccine doses (per 1 week increase)		1.19 (1.08; 1.31)^d^	0.0003	0.91 (0.82; 1.00)	0.0542
**Vaccine protocol**^**e**^			< 0.0001		< 0.0001
AST-AST	107/280 (38)	Ref		Ref	
PFI-PFI	1937/2265 (86)	9.55 (7.30; 12.5)		20.5 (10.6; 39.6)	
MOD-MOD	270/273 (99)	145.4 (45.5; 465.1)		170.7 (46.4; 627.5)	
AST-PFI^f^	140/143 (98)	90.5 (28.2; 290.8)		86.2 (26.0; 285.7)	
AST-MOD^f^	28/28 (100)				

^a^P-Value of the type 3 Wald Chi-Square test for the association between the covariate and an anti-spike IgG titer ≥ 264 BAU/mL.

^b^2872 were selected in the multivariable logistic model; 117 were deleted due to missing information on some covariates.

^c^Not included in the multivariable model because not significant in univariable analysis.

^d^Adjusted on vaccine protocol.

^e^AST = ChAdOx1 nCoV-19; MOD = mRNA-1273; PFI = BNT162b2.

^f^These two groups were combined for OR estimates.

## Discussion

Our analysis using data from a population-based multi-cohort study showed that there were strong differences in the level of anti-spike IgG titers in the study participants which were associated with several factors including age, gender, diagnosis of SARS-CoV-2 infection, vaccination date and type, and the combinations of these factors. Based on predefined profiles, the level of the anti-spike IgG was, as expected, lowest in adults with neither a vaccination nor a SARS-CoV-2 diagnosis but more strikingly, highest in those with a diagnosis of SARS-CoV-2 infection before vaccination (Fig. 1). Age was negatively associated with the level of the anti-spike IgG when there was no SARS-CoV-2 infection while it was positively associated with age in unvaccinated participants with infection at the first wave or diagnosed during follow-up (Fig. 2). In an analysis of participants who received two doses of vaccine, we found that the level of anti-spike IgG was linked to the vaccine protocol and inversely associated with age, male gender and time since the last vaccine injection. The combination of one dose of adenovirus vaccine (AST) followed by one dose of mRNA vaccine (PFI or MOD) or the combination of two-doses of MOD appeared to be associated with the strongest humoral response (Fig. 3).

Numerous studies have reported a weaker humoral response after vaccination in relation to increasing age^18–21^, while the humoral response after infection appeared to be inconsistently stronger in older individuals than in younger ones^13,22,23^. However, the level of humoral response remained significantly weaker after infection without vaccination than after vaccination without infection in all age groups except the oldest (see Supplementary Fig. S1).

A stronger immune response to moderate COVID-19^24^ or after vaccination with the BNT162b2 or mRNA-1273 vaccines^6,18,23,25,26^ has been reported in women than in men. These findings support sex-based immunological differences contributing to variations in susceptibility to infectious diseases and response to vaccines^27^. A stronger humoral response was also reported with the mRNA-1273 than with the BNT162b2 vaccine^18,28^ which results in greater vaccine effectiveness against documented infection, symptomatic COVID-19, hospitalization or ICU admission^29^. One explanation for the difference in immunogenicity observed with the different mRNA vaccine schedules could be related to the amount of mRNA used in the respective vaccines, with 30 μg in BNT162b2 and 100 μg in mRNA-1273^30^. Similarly, the SARS-CoV-2 anti-spike IgG concentrations in heterologous schedules combining a first dose of ChAdOx1 with an mRNA vaccine were clearly higher than those with the licensed two-dose ChAdOx1 vaccine schedule^31^. Finally, and consistent with other studies, vaccination following infection was associated with a high level of anti-spike IgG^32,33^ and better protection against reinfection^34^.

There is accumulating evidence of waning immunity and protection against infection after prior infection^35^, after two-doses of mRNA vaccines^6,36–38^ or after the ChAdOx1 vaccine^39^. However, the decline in antibody titers in infected subjects appears to be slower than that observed in uninfected vaccinated subjects. This finding may be related to differences in general characteristics between these two groups (median age was 48 (Q1–Q3: 41–61) years in infected *vs* 71 (Q1–Q3: 61–74) years in uninfected vaccinated subjects, P < 0.0001). It is also in line with a study showing a higher initial antibody level followed by a faster decline in uninfected subjects vaccinated with the BNT162b2 vaccine compared to patients who had been infected with the SARS-CoV-2 virus^40^. This suggests that the components of immunity that contribute to antibody persistence are different between infection and vaccination. Among potential mechanisms that could explain the differences between antibody dynamics, infected individuals have a more diverse pool of memory B cells against SARS-CoV-2 than uninfected vaccinated individuals^41^, with higher rates of maturation and more stable memory B cells population on the long term^42^.

As reported in other studies, there was a significant association between the anti-spike IgG in BAU/mL and the time between the two vaccine doses^43^, however, the association was no longer significant when adjusted for other factors.

One of the main strengths of our study is that the participants were recruited from the general population with a lower risk of selection bias than in hospital or medical cohorts. All serological analyses were performed blind to the patient’s status (infection, vaccination) and the results are highly consistent with the literature, which supports the robustness of our results.

Our study was limited by the use of a single serological method, in particular we did not measure neutralizing antibodies against the different SARS-CoV-2 variants. The estimation of the dynamics of decreasing antibody levels after vaccination was not based on an average of individual dynamics but on a single cross-section sample because no serological sample was collected at the time of vaccination. Nevertheless, our results are observed for 4 to 5 months after the second dose of vaccine and show a linear decrease in the logarithm of the titers over time. These results can be used to guide vaccination policies.

Our results support vaccination or re-vaccination with an mRNA vaccine in subjects who have been vaccinated with two doses of adenovirus vaccine, and because of the rapid decay in antibody titers after vaccination, they promote a third dose 4 to 5 months after the second in all vaccinated individuals. In individuals with a history of SARS-CoV-2 infection, 2 doses of vaccine provide effective coverage. The timeline of subsequent booster doses remains to be determined.

## Supplementary Information

Supplementary Information.

## Data Availability

In regards to data availability, data from the study are protected under the protection of health data regulation set by the French National Commission on Informatics and Liberty (Commission Nationale de l’Informatique et des Libertés, CNIL). The data can be made available upon reasonable request to the corresponding author (fabrice.carrat@iplesp.upmc.fr), after a consultation with the steering committee of the SAPRIS-SERO study. The French law forbids us to provide free access to SAPRIS-SERO data; access could however be given by the steering committee after legal verification of the use of the data. Please, feel free to come back to us should you have any additional question.
