# Supplementary material for: Heterogeneous SARS-CoV-2 humoral response after COVID-19 vaccination and/or infection in the general population
Source: Sci Rep. 2022 May 21;12:8622. doi: 10.1038/s41598-022-11787-4 (PMC9123863; doi:10.1038/s41598-022-11787-4)
Supplement: Supplementary file 1 — Supplementary Information. [file 41598_2022_11787_MOESM1_ESM.pdf]

Heterogeneous SARS-CoV-2 humoral response after COVID-19 vaccination and/or infection in the general population.

Fabrice Carrat[1]✉, Paola Mariela Saba Villarroel[2], Nathanael Lapidus[1], Toscane Fourié[2], Hélène Blanché[3], Céline Dorival[4], Jerome Nicol[4], Jean-François Deleuze[3], Olivier Robineau[4], SAPRIS-SERO study group*, Mathilde Touvier[5], Gianluca Severi[6,7], Marie Zins[8,9], Xavier de Lamballerie[2].

# Supplementary Information

## Supplementary Tables.

**Table S1**. Characteristics of participants

| Covariates | n (%) or median (Q1-Q3) |
|---|---|
| Source cohorte | |
| Constances | 6082 (44) |
| E3NE4N | 3768 (27) |
| Nutrinet-Santé | 4121 (30) |
| Age - yrs | 58 (45-71) |
| Age group (years) | |
| [20-30[ | 227 (1.6) |
| [30-40[ | 1673 (12) |
| [40-50[ | 2960 (21) |
| [50-60[ | 2455 (18) |
| [60-70[ | 2693 (19) |
| [70-80[ | 3504 (25) |
| 80+ | 459 (3.3) |
| Gender | |
| Male | 4749 (34) |
| Female | 9222 (66) |
| Living Area | |
| Rural | 2902 (21) |
| <20,000 inhab. | 2171 (16) |
| 20-000-100,000 inhab. | 2282 (17) |
| >100,000 inhab. | 6327 (46) |
| Missing | 289 |
| Household size and composition | |
| Nb adults (inc. participant) | |
| 1 | 2411 (17) |
| 2 | 6409 (46) |
| 3+ | 5150 (37) |
| Nb children (<18yrs) | |
| 0 | 10212 (73) |
| 1+ | 3758 (27) |
| Missing | 1 |
| Socio professional category | |
| Farmer | 28 (0.2) |
| Craftsmen, traders and business owners | 313 (2.3) |
| Executives and higher intellectual professions | 6666 (49) |
| Intermediate occupations | 3972 (29) |
| Employees | 2000 (15) |
| Workers | 246 (1.8) |
| Never worked | 254 (1.9) |
| Other | 145 (1.1) |
| Missing | 347 |
| Smoking status | |
| Non smoker | 6866 (50) |
| Active smoker | 1384 (10) |
| Ex-smoker | 5452 (40) |
| Missing | 269 |
| Alcohol use (in g/dy) | |
| <5 | 5642 (43) |
| [5,10[ | 2512 (19) |
| [10,20[ | 2855 (22) |
| [20,30[ | 1208 (9.2) |
| ≥30 | 901 (6.9) |
| Missing | 853 |
| BMI (kg/m$^2$) | |
| <25 | 8609 (62) |
| [25; 30[ (overweight) | 3732 (27) |
| >=30 (obese) | 1241 (8.9) |
| Missing | 389 |

**Table S1**. Characteristics of participants (continued)

| Covariates | n (%) or median (Q1-Q3) |
|---|---|
| Chronic diseases | |
| No | 9307 (67) |
| Yes (at least one) | 4552 (33) |
| Don't know | 80 (0.6) |
| Missing | 32 |
| Specific chronic diseases (a participant may suffer from several chronic diseases) | |
| Asthma, COPD, other respiratory diseases | 1781 (13) |
| Diabetes | 736 (5.3) |
| Hypertension | 1500 (11) |
| Other cardiovascular diseases | 439 (3.2) |
| Cancer | 685 (4.9) |
| Anxiety, depression | 354 (2.5) |
| Other | 2676 (19) |
| Missing | 32 |
| Month baseline sampling (2020) | |
| May | 2864 (21) |
| June | 182 (1.3) |
| July | 7662 (55) |
| August | 2462 (18) |
| September | 790 (5.7) |
| October | 11 (0.1) |
| Month follow-up sampling (2021) | |
| May | 11 (0.1) |
| June | 11711 (84) |
| July | 1836 (13) |
| August | 346 (2.5) |
| September | 49 (0.4) |
| October | 18 (0.1) |
| Infected during the 1st wave | |
| No | 11,058 (79) |
| Yes | 2913 (21) |

**Table S2.** Factors associated with an anti-spike IgG titer ≥ 264 BAU/mL at the follow-up sample

| | Anti-spike IgG ≥264 BAU/mL n (%) | P-Value |
|---|---|---|
| Follow-up profiles | | <0.0001 |
| NoVac, NoDiag | 62 (3.2) | |
| 1Vac, NoDiag | 1347 (39) | |
| 2or3Vac, NoDiag | 3974 (78) | |
| NoVac, 1Diag | 144 (13) | |
| NoVac, 2Diag | 12 (15) | |
| 1or2Vac->Diag | 24 (65) | |
| Diag->1Vac | 1198 (75) | |
| Diag->2or3Vac | 628 (93) | |
| Age group (years) | | <0.0001 |
| [20-30[ | 104 (46) | |
| [30-40[ | 626 (37) | |
| [40-50[ | 1258 (43) | |
| [50-60[ | 1203 (49) | |
| [60-70[ | 1494 (55) | |
| [70-80[ | 2433 (69) | |
| 80+ | 271 (59) | |
| Gender | | <0.0001 |
| Male | 2362 (50) | |
| Female | 5027 (55) | |
| BMI ($kg/m^2$) | | |
| <25 | 4487 (52) | 0.0229 |
| [25; 30[ (overweight) | 2045 (55) | |
| >=30 (obese) | 652 (53) | |
| Missing | *389* | |
| Smoking status before lockdown | | <0.0001 |
| Non smoker | 3635 (53) | |
| Active smoker | 655 (47) | |
| Ex-smoker | 2969 (55) | |
| Missing | *269* | |
| Alcohol use (in g/dy) | | |
| <5 | 2975 (53) | 0.3670 |
| [5,10[ | 1312 (52) | |
| [10,20[ | 1552 (54) | |
| [20,30[ | 633 (52) | |
| ≥30 | 460 (51) | |
| Missing | *853* | |
| Chronic diseases | | <0.0001 |
| Yes | 2613 (57) | |
| No | 4714 (51) | |
| Don't know | 41 (51) | |
| Missing | *32* | |
| Infected during the 1st wave | | <0.0001 |
| No | 5728 (52) | |
| Yes | 1661 (57) | |
| Time between baseline and follow-up serology (days) | | <0.0001 |
| Q1 (233-317( | 1651 (49) | |
| Q2 (317-330( | 1696 (49) | |
| Q3 (330-358( | 2204 (61) | |
| Q4 (358-514( | 1838 (53) | |

**Supplementary Figures.**

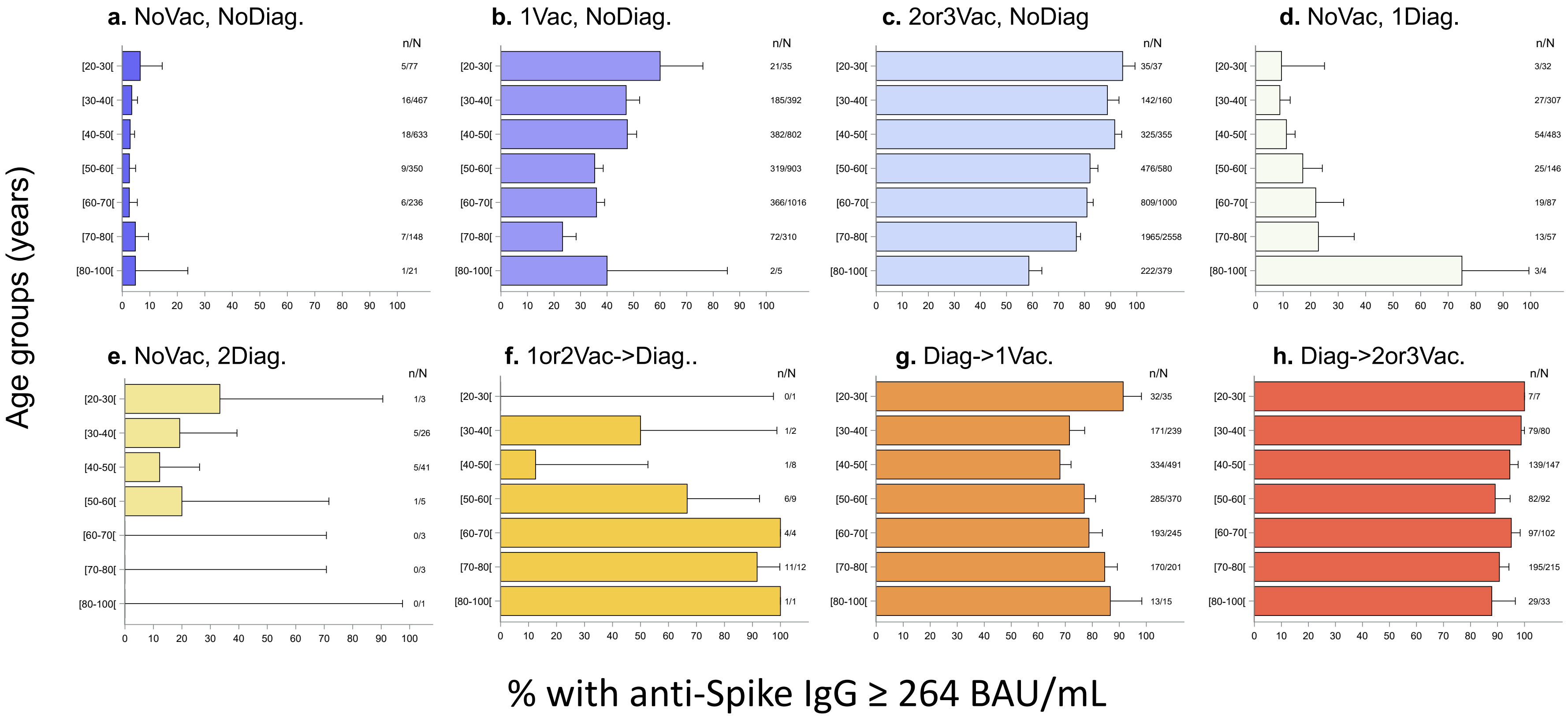


**Figure S1.** Proportion of participants with anti-spike IgG ≥ 264 BAU/mL according to age groups by follow-up profiles (**a.** to **h.**). Error bars represent upper-limit of the 95% Confidence Intervals calculated using an exact method.

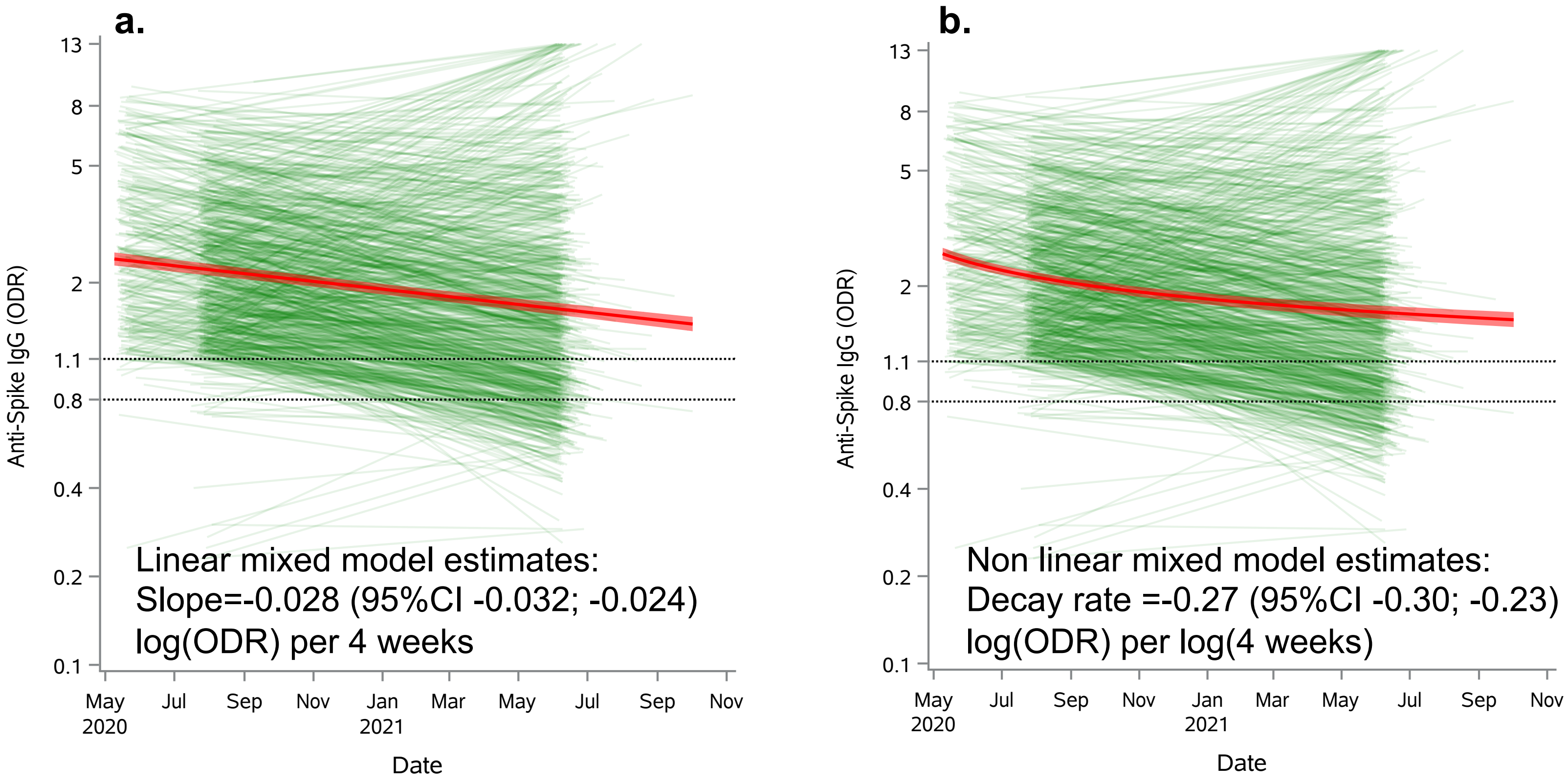


**Figure S2.** Spaghetti plot of anti-spike IgG (ODR) at baseline and follow-up in 834 participants infected during the first wave of the pandemic who remained unvaccinated at the time of the follow-up serology. **a.** Estimates from a linear mixed model with random by subject intercept. **b.** Estimates from a non-linear mixed power-law model with random by subject coefficients.

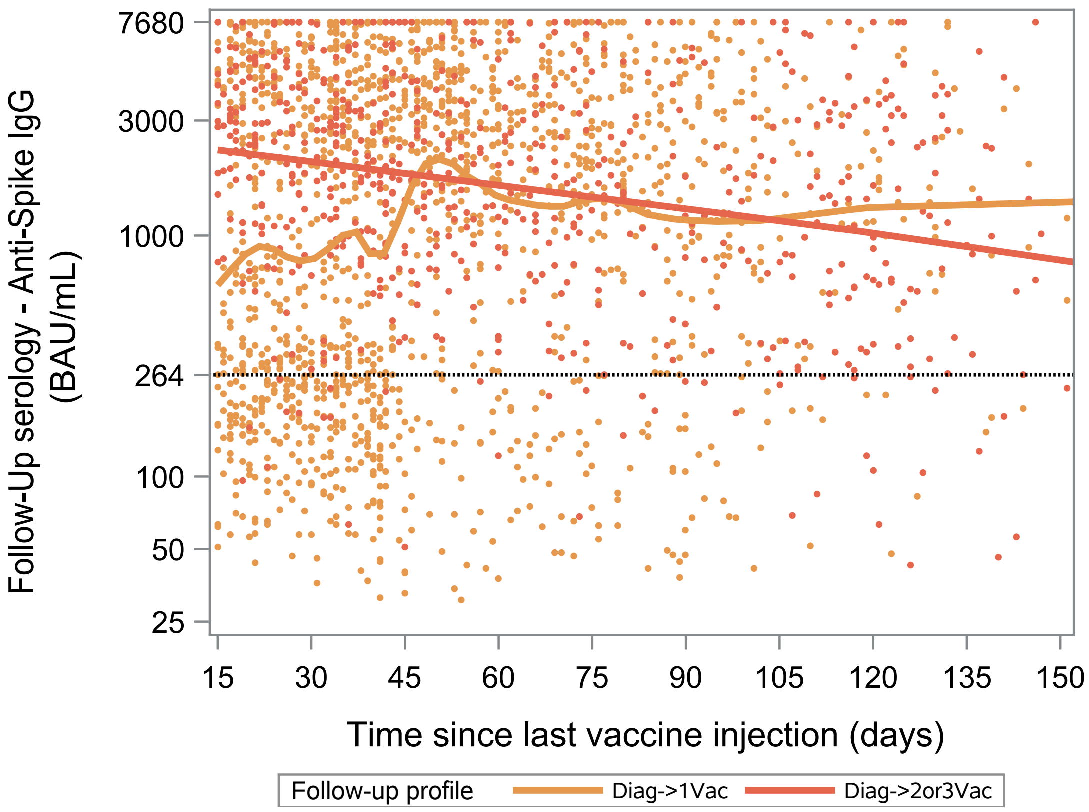


**Figure S3.** Scatter Plot of anti-spike IgG (BAU/mL) according to time since the first (Diag->1Vac) or second (Diag->2or3Vac) vaccine dose in participants with prior infection or diagnosis, and locally weighted polynomial smoothing (LOESS) trend estimates. The dashed line at 264 BAU/mL was estimated to be associated with 80% vaccine efficacy against symptomatic infection with the Alpha (B.1.1.7) variant[9]. Fourteen samples not shown, 5 participants with 3 vaccine doses not shown.

**Supplementary Note S1.**

**The SAPRIS-SERO study group (full list)**

Fabrice Carrat, Pierre-Yves Ancel, Marie-Aline Charles, Gianluca Severi, Mathilde Touvier, Marie Zins (SAPRIS-SERO investigators).
Nathalie Bajos, Fabrice Carrat, Pierre-Yves Ancel, Marie-Aline Charles, Florence Jusot, Claude Martin, Laurence Meyer, Ariane Pailhé, Alexandra Rouquette, Gianluca Severi, Alexis Spire, Mathilde Touvier, Marie Zins (SAPRIS questionnaires).
Sofiane Kab, Adeline Renuy, Stephane Le-Got, Celine Ribet, Emmanuel Wiernik, Marcel Goldberg, Marie Zins (Constances cohort).
Fanny Artaud, Pascale Gerbouin-Rérolle, Mélody Enguix, Camille Laplanche, Roselyn Gomes-Rima, Lyan Hoang, Emmanuelle Correia, Alpha Amadou Barry, Nadège Senina, Gianluca Severi (E3N-E4N cohort)
Julien Allegre, Fabien Szabo de Edelenyi, Nathalie Druesne-Pecollo, Younes Esseddik, Serge Hercberg, Mélanie Deschasaux, Mathilde Touvier (NutriNet-Santé cohort)
Marie-Aline Charles, Pierre-Yves Ancel, Valérie Benhammou, Anass Ritmi, Laetitia Marchand, Cecile Zaros, Elodie Lordmi, Adriana Candea, Sophie de Visme, Thierry Simeon, Xavier Thierry, Bertrand Geay, Marie-Noelle Dufourg, Karen Milcent (Epipage2 and Elfe child cohorts)
Delphine Rahib, Nathalie Lydie (Santé Publique France)
Clovis Lusivika-Nzinga, Gregory Pannetier, Nathanael Lapidus, Isabelle Goderel, Céline Dorival, Jérôme Nicol, Olivier Robineau, Fabrice Carrat (IPLESP – methodology and coordinating data center)
Cindy Lai, Liza Belhadji, Hélène Esperou, Sandrine Couffin-Cadiergues (Inserm)
Jean-Marie Gagliolo (Institut de Santé Publique)
Hélène Blanché, Jean-Marc Sébaoun, Jean-Christophe Beaudoin, Laetitia Gressin, Valérie Morel, Ouissam Ouili, Jean-François Deleuze (CEPH-Biobank)
Laetitia Ninove, Stéphane Priet, Paola Mariela Saba Villarroel, Toscane Fourié, Souand Mohamed Ali, Abdenour Amroun, Morgan Seston, Nazli Ayhan, Boris Pastorino, Xavier de Lamballerie (Unité des Virus Emergents)
